# Effects of renal-dose dopamine on renal tubular functions following coronary artery bypass grafting surgery

**DOI:** 10.1186/cc10955

**Published:** 2012-03-20

**Authors:** P Zeyneloglu, H Ozdemir, O Komurcu, N Bayraktar, A Sezgin, A Pirat, G Arslan

**Affiliations:** 1 Baskent University, Ankara, Turkey

## Introduction

Cardiopulmonary bypass (CPB) is regarded as an important contributor to acute kidney injury and use of renal-dose dopamine to protect the kidneys against hypoperfusion injury following cardiac surgery remains controversial. Cystatin C has been described as a sensitive biomarker of early renal tubular injury. We aimed to evaluate the effect of renal-dose dopamine on renal tubular functions in patients undergoing coronary artery bypass grafting (CABG) surgery.

## Methods

Thirty-six patients undergoing CABG surgery were prospectively randomized to receive either 2 μg/kg/minute dopamine infusion (Group D, *n *= 19) or saline as placebo (Group P, *n *= 17) starting from induction of anesthesia for 48 hours. Serial blood and urine samples after induction of anesthesia and 2, 12, 24, 48 hours post CPB were collected to measure serum cystatin C, creatinine levels and urinary β_2_-microglobulin. Intraoperative and daily measurements of hemodynamic parameters and urine output were recorded.

## Results

The groups were similar in terms of physical characteristics, perioperative hemodynamic measurements, urine outputs and surgical times. Serum cystatin C levels demonstrated similar increases during 12, 24 and 48 hours post CPB in the dopamine and placebo groups (*P *> 0.05 for all). See Table 1. No differences were detected with respect to serum creatinine and urine β_2_-microglobulin levels between the groups (*P *> 0.05 for both). GFR was preserved equally in both groups on postoperative day 2 (104.1 ± 23.1 vs. 101.4 ± 35.8 ml/minute, *P *> 0.05).

**Table 1:** Serum cystatin C levels (ng/ml) of the patients

	**Group D (***n ***= 19)**	**Group P (***n ***= 17)**	*P *value
Induction	803 ± 173	789 ± 285	0.987
2 hours CPB	857 ± 236	861 ± 347	0.664
12 hours CPB	807 ± 239	1,132 ± 396	0.052
24 hours CPB	906 ± 211	1,158 ± 432	0.149
48 hours CPB	1,296 ± 341	1,129 ± 350	0.296

## Conclusion

The results suggest that renal-dose dopamine does not exacerbate the severity of renal tubular injury when compared with the untreated controls during the early postoperative period of patients undergoing CABG surgery.
